# Pitfall in the Design and Analysis of Comparative Oncology Trials With a Time-to-Event Endpoint and Recommendations

**DOI:** 10.1093/jncics/pkac007

**Published:** 2022-02-04

**Authors:** Zachary R McCaw, Dae Hyun Kim, Lee-Jen Wei

**Affiliations:** 1 Insitro, South San Francisco, CA, USA; 2 Hinda and Arthur Marcus Institute for Aging Research, Hebrew SeniorLife, Harvard Medical School, Boston, MA, USA; 3 Harvard T.H. Chan School of Public Health, Boston, MA, USA

## Abstract

When designing a comparative oncology trial for an overall or progression-free survival endpoint, investigators often quantify the treatment effect using a difference in median survival times. However, rather than directly designing the study to estimate this difference, it is almost always converted to a hazard ratio (HR) to determine the study size. At the analysis stage, the hazard ratio is utilized for formal analysis, yet because it may be difficult to interpret clinically, especially when the proportional hazards assumption is not met, the observed medians are also reported descriptively. The hazard ratio and median difference contrast different aspects of the survival curves. Whereas the hazard ratio places greater emphasis on late-occurring separation, the median difference focuses locally on the centers of the distributions and cannot capture either short- or long-term differences. Having 2 sets of summaries (a hazard ratio and the medians) may lead to incoherent conclusions regarding the treatment effect. For instance, the hazard ratio may suggest a treatment difference whereas the medians do not, or vice versa. In this commentary, we illustrate these commonly encountered issues using examples from recent oncology trials. We present a coherent alternative strategy that, unlike relying on the hazard ratio, does not require modeling assumptions and always results in clinically interpretable summaries of the treatment effect.

When designing a typical comparative oncology study with overall or progression-free survival (PFS) as an endpoint, the median survival times are generally posited for the 2 study arms and the trial designed to detect the corresponding difference. Although it is possible to estimate the study size needed to provide adequate power for detecting such a difference, rather than using this direct approach, the median difference is routinely converted to a hazard ratio (HR) by assuming an exponential distribution of time-to-event in each arm. The study size is then determined from the hazard ratio. The reason for taking this detour is that the estimated median is generally unstable; thus, the study size needed to detect a median difference can be impractically large. Moreover, the median overall or PFS provides only a local summary of the survival curve, which cannot capture the short-term or long-term profile (1,2). If the treatment effect is late-occurring or affects only long-term survival, which may not be known at the study design stage, then an analysis based on the median difference may lack power to detect it. On the other hand, although the hazard ratio provides a global comparison of 2 survival curves, it lacks a clear clinical interpretation, particularly when the proportional hazards assumption is not met (1,3). This creates a dissonance where the hazard ratio is used for the formal design and analysis while the median is used descriptively for interpretation.

One drawback to using the hazard ratio to design an event-driven study is that we cannot control the study duration, because having adequate power mainly depends on the total number of observed events rather than the duration of follow-up. If the event rate is high, the study duration may be short, providing insufficient information regarding the therapies’ safety profiles. On the other hand, if the event rate is low, the study duration may be long. This is burdensome to patients and investigators, and the investigated therapy may become obsolete by the study’s completion. Thus, even when using the hazard ratio to design the study, one may need to prespecify a clinically meaningful follow-up period that depends not only on the observed number of events to adequately evaluate both efficacy and safety.

When analyzing data at the end of the trial, conclusions regarding treatment efficacy are typically based on hazard ratio analysis. To provide a clinical interpretation, the observed medians are also reported descriptively but without formal comparisons, making it unclear whether their difference is statistically meaningful. The hazard ratio and the median difference provide very different contrasts of 2 time-to-event distributions, and a statistically significant difference via the hazard ratio does not imply a statistically significant difference in medians or vice versa. This creates a dissonance where the primary analysis, based on a hazard ratio, may suggest a treatment difference that completely lacks support from an interpretable summary measure, such as the median difference. Another complication is that the population median may not be estimable for a study with short-term follow-up. When the proportional hazards assumption does not hold, it is unclear how to interpret the resulting hazard ratio (1-3). For this case, a weighted log-rank test or a test based on a nonproportional hazards model, such as a model that includes time-varying effects, may be used to summarize the treatment effect in terms of a *P* value (4). However, these statistical tests do not provide estimates for the size of the treatment effect. A *P* value alone cannot be used to quantify clinical utility (5). Moreover, even when the proportional hazards assumption is plausible, it is not straightforward to interpret a hazard ratio of, for example, 0.75 in favor of the new treatment. This ratio does not mean that the treatment reduces the risk of mortality by 25% relative to the reference because hazard is not a probability measure like risk. Rather, hazard is the “force of mortality,” which is an intensity rate measure and is difficult to estimate well without modeling. Additionally, as a relative measure, the hazard ratio alone lacks context without a hazard curve from the reference arm across the entire study period. If the underlying hazard is low, then a 25% reduction in hazard relative to the reference arm may not be clinically meaningful.

When the study results from the hazard ratio are quite different from those based on the median difference, the trial may provide a conclusion about the treatment effect based mainly on the hazard ratio’s *P* value. After investing tremendous resources to conduct the trial, we are left without knowing how to quantify and interpret the clinical utility for the study therapies.

Numerous recent trials have encountered the pitfall of using separate summary measures for the design and analysis vs the interpretation of results (6–11). As a specific example, consider KEYNOTE-604, which compared pembrolizumab and placebo among patients with extensive-stage small-cell lung cancer (10). The primary endpoint was PFS. The study was designed assuming medians of 4.3 and 6.6 months for PFS with placebo and pembrolizumab. Assuming exponential models, the hazard ratio is 0.65 on conversion. Using data reconstructed (12) from the original publication (10), the PFS curves are presented in Figure 1, A below. Because the Kaplan-Meier (KM) curves are intertwined across the first 4 months, the proportional hazards assumption was clearly not met. Consequently, the reported hazard ratio of 0.75 is not clinically interpretable. Note that as mentioned before, we cannot conclude that the risk of progression was 25% lower among patients receiving pembrolizumab, because hazard is not a direct measure of risk (1–3). The observed medians for PFS were 4.3 and 4.5 months for pembrolizumab and placebo, respectively. Although no formal comparison of medians was presented in the publication, based on reconstructed data, the 95% confidence interval for the difference of medians was −0.6 to 0.8 months, providing no evidence for a difference between the population medians. This is an example where conventional inference about the treatment difference would be primarily based on a *P* value that lacks support from clinically interpretable summary measures.

**Figure 1.**
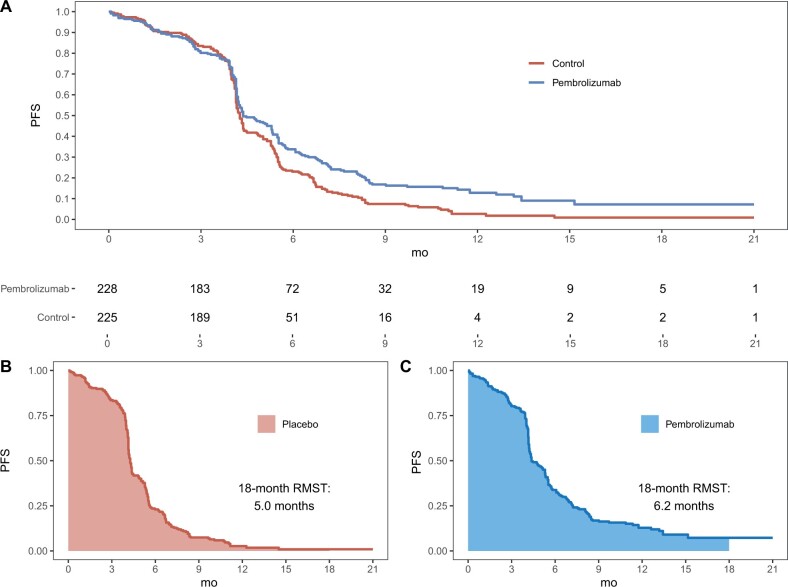
The 18-month restricted mean survival time (RMST) analysis of progression-free survival (PFS) from the KEYNOTE-604 study. **A**) Reconstructed PFS curves comparing pembrolizumab or placebo, in addition to etoposide and platinum, for patients with extensive-stage small-cell lung cancer are shown. The numbers of patients at risk are shown below the graph. The 18-month restricted mean PFS times as the area under the PFS curve for **B**) placebo and **C**) pembrolizumab are shown.

Instead of using medians to justify the choice of hazard ratio at the design stage, trialists sometimes specify survival rates at a landmark timepoint, expressing the target treatment effect as a rate difference. However, rather than designing the study via this difference, the study size is typically estimated by converting the assumed survival rates to a hazard ratio. This was the strategy taken in the recent Adjuvant Platinum and Taxane in Triple-negative Breast Cancer trial exploring whether a paclitaxel plus carboplatin regimen (PCb) would improve disease-free survival (DFS) relative to anthracycline and docetaxel (CEF-T) among women with triple-negative breast cancer (11). From the DFS KM curves presented in the publication, the proportional hazards assumption was likely not met, making it difficult to interpret the observed hazard ratio of 0.65. Moreover, the median was not reached in either arm, and the 5-year DFS rates of 86.5% for PCb and 80.3% CEF-T were presented without formal comparison. As with KEYNOTE-604, it is unclear how to appropriately quantify the size of the PCb benefit from this study.

From the above examples, having separate summaries for evaluating and interpreting the treatment difference is suboptimal and may result in incoherent conclusions. Ideally, one would use a single, clinically interpretable, and global summary of the event-time profile as the primary estimand, whose estimate is efficient for detecting a clinically meaningful treatment effect. So, the question is whether there are alternatives to the median/hazard ratio approach for the design and analysis of oncology trials.

Now, from Figure 1, A, the higher the KM curve, the better the therapy. Therefore, a larger area under the curve suggests better treatment efficacy. In fact, the area under the curve up to the specific time window is the *t*-year restricted mean survival time (RMST), which is the expected survival time across *t* years of follow-up (1–3). For example, using reconstructed PFS data from KEYNOTE-604 (8), the 18-month survival times were 6.2 months for pembrolizumab and 5.0 months for placebo, which are displayed in Figure 1, B and C. The difference of 1.2 months (95% CI = 0.5 to 1.9, *P* = .001) is highly statistically significant in favor of pembrolizumab. This single, interpretable summary provides both statistical and clinical evidence of treatment efficacy. Specifically, across 18 months of follow-up, patients receiving pembrolizumab are expected to survive an additional 1.2 months on average. Because mean survival time analysis requires no modeling assumptions, this interpretation is always valid. Notice that the PFS KM nearly reached zero by the end of follow-up. When this occurs, the restricted mean survival time, over 18 months in the present case, will be close to the (unrestricted) mean survival time across the patients’ entire lives. Using parametric Weibull models fit to the observed data, the estimated mean survival times were likewise 6.2 months for pembrolizumab and 5.0 months for placebo, with a difference of 1.2 months. Such parametric modeling allows investigators to extrapolate the expected treatment difference if follow-up were continued indefinitely.

For Adjuvant Platinum and Taxane in Triple-negative Breast Cancer (11), using reconstructed data from the publication, the 10-year (120-month) mean survival times were 107 months for PCb and 101 months for CEF-T. Note that 10 years is the period over which the log-rank test in the publication was calculated. The difference of 6.0 months (95% CI, 0.4 to 11.6, *P* = .036) favors PCb.

The need to specify a truncation time *t* is often cited as a limitation of the RMST, and inferences concerning the treatment effect can depend on its choice (13,14). If the study does not have a prespecified time window for analysis, the hazard ratio analysis is valid only up to the largest observed event time from both arms (15). On the other hand, the RMST can generally be estimated until the last observed event or censoring time (15). Therefore, RMST analysis utilizes more empirical information than its hazard ratio counterpart. As mentioned before, the study duration should ideally be prespecified at the design stage based on safety and efficacy considerations. Sensitivity analyses can be conducted for various choices of truncation time. As with other robust statistical procedures, a limitation of the RMST analysis is that it may not be as efficient as the hazard ratio estimation when the proportional hazards assumption in fact holds. On the other hand, when this model assumption is not met, the estimated hazard ratio is not a simple average of hazard ratios over time (16,17). For this case, the RMST can provide a substantial power advantage (18). Methodology and software to perform the power and sample size calculations are publicly available (2).

All analytic methods in survival analysis have advantages and disadvantages, and no single method is superior in all circumstances. On the other hand, effect size estimates provide more useful and actionable information regarding treatment efficacy than hypothesis tests alone (19). Although no single summary measure can capture all information contained in the survival profile, we need a single clinically relevant summary for designing the trial, quantifying treatment efficacy, and facilitating decision making. In a comparative study, by considering the entire survival profile, the hazard ratio and *t*-year mean survival time can increase statistical power. Yet in contrast to the hazard ratio, the mean survival time makes no modeling assumptions and is always estimable and interpretable (20). To make the design and analysis of clinical trials more coherent, we encourage trialists to move beyond the conventional framework when conducting future oncology studies.

## Funding

Not applicable.

## Notes

**Role of the funder:** Not applicable.

**Author disclosures:** The authors have no disclosures.

**Author contributions:** LJW: conceptualization, writing—original draft, writing—review & editing. ZRM: data curation, formal analysis, software, visualization, writing—original draft, writing—review & editing. DHK: writing—original draft, writing—review & editing.

## Data Availability

No new data were generated in this manuscript. The data analyzed were reconstructed from the published Kaplan-Meier curves in the original articles using the method of Guyot et al. (12)
